# Risk of Shingles in Adults with Primary Sjogren’s Syndrome and Treatments: A Nationwide Population-Based Cohort Study

**DOI:** 10.1371/journal.pone.0134930

**Published:** 2015-08-25

**Authors:** Jen-Yin Chen, Li-Kai Wang, Ping-Hsun Feng, Chin-Chen Chu, Tain-Junn Cheng, Shih-Feng Weng, Su-Zhen Wu, Tsung-Hsueh Lu, Chia-Yu Chang

**Affiliations:** 1 Department of Anesthesiology, Chi Mei Medical Center, Tainan, Taiwan; 2 Department of the Senior Citizen Service Management, Chia Nan University of Pharmacy and Science, Tainan, Taiwan; 3 Department of Neurology, Chi Mei Medical Center, Tainan, Taiwan; 4 Department of Medical Research, Chi Mei Medical Center, Tainan, Taiwan; 5 Institute of Public Health, College of Medicine, National Cheng Kung University, Tainan, Taiwan; 6 Department of Food Science and Applied Biotechnology, National Chung Hsing University, Taichung, Taiwan; 7 The center for General Education, Southern Taiwan University of Science and Technology, Tainan, Taiwan; University of California Riverside, UNITED STATES

## Abstract

**Background:**

Primary Sjögren's syndrome (pSS) is associated with immunological dysfunctions—a well-known risk factor of shingles. This study aimed to examine the incidence and risk of shingles in adults with pSS and pharmacological treatments.

**Methods:**

This retrospective population-based cohort study was conducted using National Health Insurance claims data. Using propensity scores, 4,287 pSS adult patients and 25,722-matched cohorts by age, gender, selected comorbidities and Charlson comorbidity index scores were identified. Kaplan-Meier analysis and Cox regression were conducted to compare the differences in developing shingles. In pSS, oral and eye dryness are treated with substitute agents. Extraglandular features are often treated with pharmacological drugs including steroids and immunosuppressants. pSS patients were grouped as follows: no pharmacological drugs, steroids alone; immunosuppressants alone; combined therapies.

**Results:**

During the follow-up, 463 adults with pSS (10.80%) and 1,345 control cohorts (5.23%) developed shingles. The cumulative incidence of shingles in pSS patients (18.74/1,000 patient-years) was significantly higher than controls (8.55/1,000 patient-years). The adjusted hazard ratio (HR) of shingles was 1.69 (95% confidence interval (CI) 1.50–1.90). In age-subgroup analyses, incidences of shingles in pSS increased with age and peaked in pSS patients aged ≧60; however, adjusted HRs decreased with age. Compared to control cohorts with no drugs, adjusted HRs for shingles in pSS patients were ranked from high to low as: combined therapies (4.14; 95% CI 3.14–5.45) > immunosuppressants alone (3.24; 95% CI 2.36–4.45) > steroids alone (2.54; 95% CI 2.16–2.97) > no pharmacological drugs (2.06; 95% CI 1.76–2.41). Rates of shingles-associated hospitalization and postherpetic neuralgia were 5.62% and 24.41%, both of which were significantly higher than those (2.60%; 13.01%) in the control cohorts.

**Conclusions:**

Adults with pSS were at greater risk for shingles than control cohorts. Drug exposures significantly increased the risk of shingles in pSS.

## Introduction

Primary Sjögren’s syndrome (pSS) is an autoimmune disorder of the exocrine glands with associated lymphocytic infiltrates of the affected glands characterized with oral and eye dryness [1,2]. Other than sicca symptoms, there is often systemic involvement (extraglandular manifestations). Substitute agents (sialogogues and eyedrops) are standard management for controlling sicca symptoms. Extraglandular features are often treated with pharmacological drugs including steroids and immunosuppressants [3]. Furthermore, people with pSS are found to have a lower intake of vitamin C [4]. Taken together, people with pSS may be at increased risk of infections due to dysfunctions of the immune system, pharmacological treatments and altered nutrient intakes [3–6].

Herpes zoster (shingles) is a common viral disease caused by reactivation of latent varicella zoster virus which leads to painful vesicular eruptions in a dermatomal distribution. Usually shingles and herpetic pain subsides spontaneously. Approximately 13–25% of shingles patients suffer from herpetic pain lasting 3 months or longer, which is defined as postherpetic neuralgia [7]. Postherpetic neuralgia, the most common complication of shingles, often leads to a profoundly negative impact on one’s quality of life [7–9]. Because of the substantial financial and health care burden imparted by shingles and its complications, elucidation of risk factors associated with this illness is important [6,10–13]. Interestingly, pSS along with rheumatoid arthritis and systemic lupus erythematosus (SLE) are the three most common autoimmune diseases [14–16]. The associations between shingles and rheumatoid arthritis/SLE have been reported in many medical articles [10–13]. However, the association between pSS and shingles is under studied: a keyword search in PUBMED on the relationship of pSS and shingles returned zero publications. To our knowledge, only one study examined the associations between pSS and shingles [17]. The finding that pSS patients are not at risk of shingles is questionable due to the flawed design of that study. In particular, the associations between the risk of shingles and pharmacological treatments of pSS including steroids and immunosuppressants have not been examined. Considered an unmet requisite in pSS research [16], the goal of this study was to estimate the incidence of adults with pSS developing shingles using nationwide population-based cohorts in Taiwan. Furthermore, the incidence of developing shingles and shingles-associated complications in adults with pSS exposed to different pharmacological treatments was also assessed.

## Methods

### Data source

This retrospective cohort study used the National Health Insurance Research Database (NHIRD) of Taiwan which contains approximately 23 million members. The Taiwan National Health Insurance program is a health care system established in 1995. It is a mandatory single-payer health insurance system in which all citizens are obliged to participate. The coverage rate in 2008 was approximately 99.5% of Taiwan’s entire population. The claims data are generally accurate. Thus far, more than 1000 studies based on these data have been published in peer-reviewed journals [18,19]. In this study, we used Taiwanese longitudinal NHIRD from 1996–2011.

The Bureau of National Health Insurance specifies 30 categories of catastrophic illnesses. These illnesses include cancers, congenital illnesses, end-stage renal disease, and several autoimmune diseases such as pSS. In Taiwan, a patient suffering from any catastrophic illness can apply for a catastrophic illness certificate. To obtain a catastrophic illness certificate, the patient’s attending rheumatologist is required to provide relevant clinical and laboratory information as part of the application for review. The review committee then assesses the application according to the specific criteria for each diagnosis. A patient is given a catastrophic illness certificate and exempted from co-payment if approved. Hence, the accuracy of the pSS diagnosis was not a concern in this study [1,2].

### Cohort of adults with pSS

We identified 8,423 adult patients (≧ 20 years old) with ICD-9-CM 710.2 and approval of the catastrophic illness certificate for pSS between 2001 and 2008. Exclusion criteria included (1) patients with secondary Sjogren’s syndrome who had a catastrophic illness certificate for Sjogren’s syndrome and a catastrophic illness certificate for any other autoimmune disease, such as SLE, rheumatoid arthritis and/or other connective tissue diseases [1,2,20,21]; (2) patients with lymphoma, sarcoidosis, hepatitis C viral infection, organ transplants, human immunodeficiency virus infection, chronic obstructive pulmonary disease or those who have experienced radiotherapy to the head and/or neck, [1,2,14,22,23]; (3) patients with pSS had been diagnosed with shingles previously; and (4) unmatchable patients. Anyone meeting the aforementioned exclusion criteria was excluded from the study because these patients may have been treated with steroids and immunosuppressants or suffered from immunosuppressive conditions which are potential confounders of shingles. As a result, 4,287 adult patients with pSS were identified as study cohorts (S1 Fig).

The controls were matched with adult pSS patients by age, gender, selected comorbidities and Charlson comorbidity index (CCI) scores by propensity scores from the remaining database patients without pSS diagnosis [24].

### Drug exposures

Medications were coded according to the anatomical therapeutic chemical drug classification system. The current medications for pSS include substitutive agents (artificial tears, salivary substitutes) and pharmacologic therapies (steroids, immunosuppressants and biologic agents) [3]. Immunosuppressants include methotrexate, azathioprine, leflunomide, tacrolimus, sirolimus, cyclosporine, cyclophosphamide, hydroxychloroquine, sulfasalazine, gold thiomalate, mycophenolic acid, mycophenolate mofetil, interferons, D-penicillamine and thalidomide [3]. During the study period in Taiwan, available biologic agents for pSS included etanercept and adalimumab which could be prescribed alone or prescribed as combined therapies with steroids and/or immunosuppressants. In total, there was a small number (<25) of pSS patients prescribed biologic agents. Thus, pSS patients prescribed biologic agents were grouped into “immunosuppressants group” rather than creating more subgroups which were too small to provide significant information. As a result, drug-subgroup analysis for the treatment of pSS was collected with a focus on steroids and immunosuppressants. For the comparison, pSS patients were grouped as follows: (1) No drugs (no steroids/immunosuppressants); (2) Steroids alone; (3) Immunosuppressants alone; (4) Combined therapies of steroids and immunosuppressants. The impact of medications stratified by age groups was also studied. Adult patients with pSS were divided into two age groups: 20~49 and ≧50.

Based on the assumption that shingles is an acute event, only recent drug exposure was considered to be related to the incident shingles. A recent exposure was defined as drugs prescribed at the time of the event or within the previous 90 days for 3 days or at least 3 days prior to the outbreak [11,18,25,26].

### Identification of shingles

Incident shingles cases were identified by an automatch search for codes of shingles and zoster complications (ICD-9 codes 053.0–053.9) listed on the outpatient/inpatient diagnosis claim data. Patients receiving a diagnosis of shingles before the date of cohort entry were excluded from both the study and control cohorts. Since the date of cohort entry, all patients were followed until the development of shingles or until the end of 2011, whichever was earliest [18].

### Complications of shingles

We examined postherpetic neuralgia and nonpain complications among patients suffering from shingles. Postherpetic neuralgia was either identified with post-herpetic neuralgia ICD-9-CM 053.1 or defined as visiting a physician with the shingles codes lasting more than 90 days and receiving pain medications/treatment for neuralgia [19,27]. Nonpain complications included ophthalmic zoster (053.2), neurological, dermatological and other shingles-related complications [7,18,19,27,28] (S1 Appendix).

### Statistical analysis

Data processing and statistical analysis were performed using SAS statistical software (Version 9.3.1; SAS Institute, Cary, NC, USA). Chi-square tests were used to test the differences in categorical variables between the pSS patients and the control cohorts. The difference of continuous variables between the two groups was performed by Student's t-test. Kaplan-Meier analysis was used to calculate the cumulative hazard rates of shingles between the two groups and the log-rank test was used to test the difference between the survival curves. A Cox regression model was performed to compare the adjusted hazard ratio (HR) of shingles between the pSS and control cohorts by adjusting for age group, selected comorbidities, Charlson comorbidity index scores, drugs, monthly income and geographical region. A *p* value of <0.05 was considered significant [18].

### Ethics

This retrospective study was approved by the Institutional Review Board of the Chi Mei Medical Center, Tainan, Taiwan. Written informed consent was waived because we used de-identified secondary data released to the public for research purposes.

## Results

We identified 4,287 adults diagnosed with pSS and 25,722 controls with a ratio of 1:6. The distributions of demographic characteristics, selected comorbidities and CCI scores for adults with pSS and the control cohorts are shown in Table 1. No statistically significant differences were noted between adults with pSS and control cohorts (adults without pSS) regarding age/gender distribution, selected comorbidities and CCI scores because we used propensity-score matching. The female to male ratio was 6:1 with females being predominant.

**Table 1:** Demographic characteristics for pSS adults and the control cohorts in Taiwan between 2001 and 2008.

	Patients with pSS	Control cohorts	
	N = 4,287	N = 25,722	*P*
Variable	Number (%)	Number (%)	
Age, years			
20–29	150 (3.50)	887 (3.45)	0.410
30–39	443 (10.33)	2,497 (9.71)	
40–49	891 (20.78)	5,135 (19.96)	
50–59	1,185 (27.64)	7,255 (28.21)	
≧60	1,618 (37.74)	9,948 (38.68)	
Average age, mean (SD) years	55.32 (14.03)	55.66 (13.91)	0.136
Gender			
Female	3,656 (85.28)	22,126 (86.02)	0.198
Male	631 (14.72)	3,596 (13.98)	
Comorbidities			
Malignancy	158 (3.69)	929 (3.61)	0.811
Diabetes mellitus	332 (7.74)	2,088 (8.12)	0.406
Hypertension	954 (22.25)	6,006 (23.35)	0.115
Charlson comorbidity index score			
0	3,270 (76.28)	19,593 (76.17)	0.881
≧1	1,017 (23.72)	6,129 (23.83)	

pSS: primary Sjogren’s syndrome; SD: standard deviation.

Categorical variable was estimated by chi-square test and continuous variable was estimated by student’s t-test.

*P* < 0.05, statistical significance.

### Incidence of shingles

**Fig 1:**
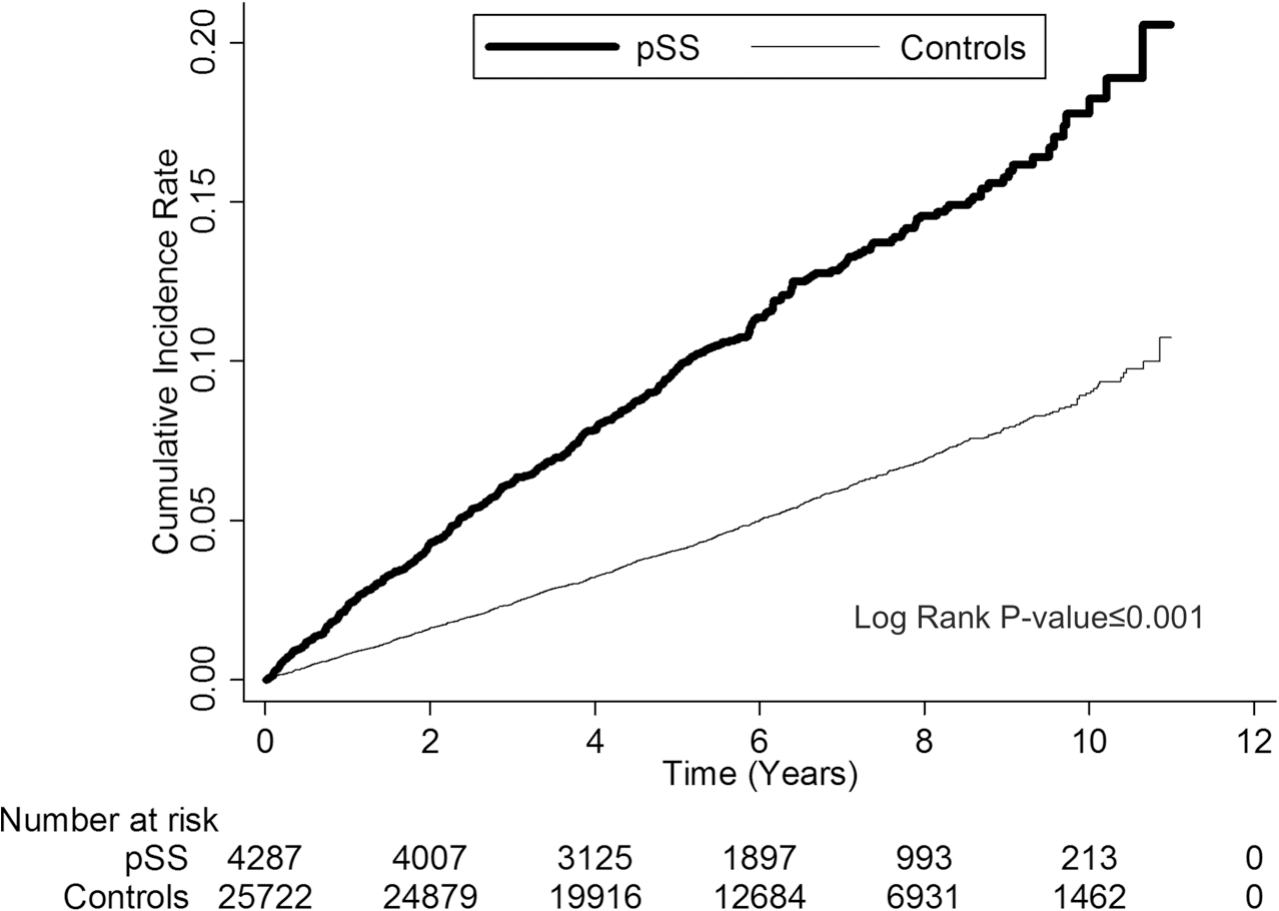
Cumulative incidences of shingles for pSS adults and the control cohorts from 2001 to 2011.

In total, 463 adults with pSS (10.80%) and 1,345 control cohorts (5.23%) were diagnosed as having shingles during the follow-up period (*P*<0.001). The overall incidence of shingles for pSS adults and the control cohorts were 18.74 and 8.55/1000 patient-years, respectively (Table 2). Cox regression analysis revealed that pSS is an independent risk factor for shingles after adjusting for potential confounders with adjusted HR of 1.69 (95% confidence interval (CI), 1.50–1.90). The cumulative incidence of shingles in pSS adults was significantly higher than that of the control cohorts over the follow-up period (*P*<0.001) (Fig 1).

**Table 2:** Incidence of shingles for pSS adults *vs*. the control cohorts during the 11-year follow-up.

All subjects	Total	Shingles	Person-years	Incidence per	Crude HR	Adjusted HR^a^
(N = 30,009)	Number	Number	at risk	1000 person-years	(95% CI)	(95% CI)
Control	25,722	1,345	157,353	8.55	1.00	1.00
pSS	4,287	463	24,712	18.74	2.20* (1.98–2.44)	1.69* (1.50–1.90)

pSS: primary Sjogren’s syndrome; HR: hazard ratio; CI: confidence interval.

^a^ Data are adjusted by age group, gender, comorbidities (malignancy, diabetes mellitus and hypertension), Charlson comorbidity index score, drugs, geographic region and income.

*P <0.05 for statistical significance.

### Age/gender subgroup analyses for incidence of shingles

In age-subgroup analyses, age-specific incidence for shingles in pSS increased with age resulting in the highest incidence of shingle in those aged 60 and older (Table 3). Adjusted HRs of shingles in pSS adults compared with the control cohorts reached statistical significance in all of the age-subgroups. Interestingly, the adjusted HRs of shingles in pSS adults compared with the control cohorts peaked in those aged 20 to 29 and hit the lowest level in those aged 50 and older. As for gender-subgroup analyses, both male and female adults with pSS had greater risks of developing shingles than the control cohorts.

**Table 3:** Age/gender subgroup analysis for incidence of shingles in pSS adults during the follow-up.

	Primary Sjogren’s syndrome				Control cohorts					
	Total number	Shingles cases	Person-years at risk	Incidence per 1000 person-years	Total number	Shingles cases	Person-years at risk	Incidence per 1000 person-years	Adjusted HR (95% CI)	*P*
**Age, years**										
20–29	150	9	887	10.15	887	12	5,409	2.22	4.22 (1.72–10.35) ^a^	0.002
30–39	443	20	2,681	7.46	2,497	29	15,605	1.86	3.98 (2.24–7.05) ^a^	<0.001
40–49	891	84	5,336	15.74	5,135	182	32,536	5.59	2.79 (2.16–3.62) ^a^	<0.001
50–59	1,185	128	6,748	18.97	7,255	411	43,834	9.38	2.05 (1.68–2.50) ^a^	<0.001
≧60	1,618	222	9,061	24.50	9,948	711	59,968	11.86	2.07 (1.78–2.41) ^a^	<0.001
**Gender**										
Male	631	71	3,690	19.24	3,596	194	22,244	8.72	2.26 (1.72–2.96) ^b^	<0.001
Female	3,656	392	21,022	18.65	22,126	1,151	135,109	8.52	2.24 (2.00–2.51) ^b^	<0.001

HR: hazard ratio; CI: confidence interval.

^a^ Data are adjusted by gender, comorbidities (malignancy, diabetes mellitus and hypertension), Charlson comorbidity index score, drugs, geographic region and income.

^b^ Data are adjusted by age, comorbidities (malignancy, diabetes mellitus and hypertension), Charlson comorbidity index score, drugs, geographic region and income.

*P* <0.05, statistical significance.

### Pharmacological treatments and the incidence of shingles

The control cohorts with no drugs were used as the reference group. Compared to the reference group, the adjusted HRs for shingles in pSS adults with and without drugs (steroids and/or immunosuppressants) were 2.06 (95% CI 1.76–2.41) and 2.84 (95% CI 2.48–3.25), respectively (*P* < 0.001; *P* < 0.001). Furthermore, compared to pSS adults without drugs, the adjusted HRs for shingles in pSS adults receiving drugs was 1.40 (95% CI 1.16–1.69) (*P* < 0.001), indicating that drug exposures in pSS adults significantly increased the risk of shingles (Table 4). The impact of medications stratified by age groups, in pSS adults aged ≧50, the risk of shingles in pSS adults receiving drugs was significantly greater than that for pSS adults without drugs (adjusted HR 1.43, 95% CI 1.15–1.78) (*P* = 0.001). However, in pSS adults aged 20~49, there was no significant difference in the risk between patients with and without drugs (*P* = 0.159). Based on the pharmacological therapies of pSS, the adjusted HRs for shingles in pSS adults compared to the reference group were ranked from high to low as: Combined therapies of steroids and immunosuppressants > Immunosuppressants alone > steroids alone > no drugs. (all *P* < 0.001) In addition, compared to pSS patients without drugs, adjusted HRs of shingles in all subgroups of pSS patients receiving pharmacological treatments reached statistical significance (all *P* < 0.05). Adjusted HRs of shingles peaked in those receiving combined therapies of steroids and immunosuppressants (2.06, 95% CI 1.52–2.79) and hit the lowest in those receiving steroids alone (1.27, 95% CI 1.03–1.56). These results suggest that the incidence of shingles in pSS adults varied with different pharmacological exposures.

**Table 4:** Impact of pharmacological therapies on incidence of shingles for pSS adults and the control cohorts during the follow-up.

			Total number	Shingles cases	Person-years at risk	Incidence per 1000 person-years	Adjusted HR (95% CI)	*P*	Adjusted HR (95% CI)	*P*
**(A) Overall impact of medications**										
	Control cohorts (No steroids/immunosuppressants)		21,152	976	128,378	7.60	1.00		X	
	Primary Sjogren’s syndrome									
		No drugs (No steroids/immunosuppressants)	2,177	189	12,366	15.28	2.06 (1.76–2.41)^a^	<0.001	1.00	
		Drugs (Steroids and/or immunosuppressants)	2,110	274	12,347	2.19	2.84 (2.48–3.25)^a^	<0.001	1.40 (1.16–1.69)^a^	<0.001
**(B) The impact of medications, stratified by age groups**										
Aged 20–49										
	Control cohorts (No steroids/immunosuppressants)		7,418	167	46,111	3.62	1.00		X	
	Primary Sjogren’s syndrome									
		No drugs	813	52	4,772	10.90	3.04 (2.22–4.15)^b^	<0.001	1.00	
		Drugs	671	61	4,131	14.77	3.96 (2.95–5.31)^b^	<0.001	1.31 (0.90–1.90)^b^	0.159
Aged 50 and older										
	Control cohorts (No steroids/immunosuppressants)		13,734	809	82,266	9.83	1.00		X	
	Primary Sjogren’s syndrome									
		No drugs	1,364	137	7,594	18.04	1.83 (1.53–2.20)^b^	<0.001	1.00	
		Drugs	1,439	213	8,216	25.93	2.62 (2.25–3.04)^b^	<0.001	1.43 (1.15–1.78)^b^	0.001
**(C) The impact of medications, stratified by the usage of drugs**										
	Control cohorts (No steroids/immunosuppressants)		21,152	976	128,378	7.60	1.00		X	
	Primary Sjogren’s syndrome									
		No drugs (No steroids/immunosuppressants)	2,177	189	12,366	15.28	2.06 (1.76–2.41)^a^	<0.001	1.00	
		Steroids alone	1,471	180	8,748	20.58	2.54 (2.16–2.97)^a^	<0.001	1.27 (1.03–1.56)^a^	0.023
		Immunosuppressants alone	335	40	1,877	21.31	3.24 (2.36–4.45)^a^	<0.001	1.53 (1.08–2.15)^a^	0.016
		Combined therapies (Steroid + Immunosuppressants)	304	54	1,721	31.37	4.14 (3.14–5.45)^a^	<0.001	2.06 (1.52–2.79)^a^	<0.001

HR: hazard ratio; CI: confidence interval.

^a^ Data are adjusted by age group, gender, comorbidities (malignancy, diabetes mellitus and hypertension), Charlson comorbidity index score, geographic region and income.

^b^ Data are adjusted by gender, comorbidities (malignancy, diabetes mellitus and hypertension), Charlson comorbidity index score, drugs, geographic region and income.

*P* <0.05, statistical significance.

### Outcomes of herpes zoster

The hospitalization rate for herpes zoster was 5.62% in pSS adults, which was higher than that in the control cohorts (2.60%) (*P*<0.001). The rate of postherpetic neuralgia in pSS adults with herpes zoster was 24.41%, which was higher than that in the control cohorts (13.01%) (*P*<0.001). However, there were no significant differences in the rates of nonpain complications between pSS adults and the control cohorts. Overall, adult pSS patients were at significantly greater risks of shingles-related hospitalization and postherpetic neuralgia than the control cohorts (Table 5).

**Table 5:** Outcomes of shingles in pSS adults *vs*. the control cohorts.

	Primary Sjogren’s syndrome	Control cohorts	*P*
	Number (%)	Number (%)	
Shingles-related hospitalization	26 (5.62)	35 (2.60)	<0.001
Shingles complications			
Postherpetic neuralgia	113 (24.41)	175 (13.01)	<0.001
Nonpain complications			
Ophthalmic shingles	23 (4.97)	51 (3.79)	0.27
Neurologic complications	47 (10.15)	120 (8.92)	0.43
Dermatological complications	57 (12.31)	173 (12.86)	0.76
Others	17 (3.67)	34 (2.53)	0.20

Categorical variable was estimated by chi-square test.

*P* < 0.05, statistical significance.

## Discussion

To our knowledge, this is the first nationwide population-based cohort study to explore the risk of shingles in adults with pSS. Incidence of shingles among adults with pSS was 1.69 times higher than adults without pSS. Age-subgroup analyses showed that age-specific incidence increased with age; however, the adjusted HR decreased with age. No significant differences in incidence and adjusted HR by gender were noted. Compared to the control cohorts with no steroids and no immunosuppressants (7.60/1000 patient-years), pSS adults receiving no pharmacological drugs revealed the lowest incidence (15.28/1000 patient-years) and pSS adults with combined therapies of steroids and immunosuppressants demonstrated the highest incidence (31.37/1000 patient-years). Overall, drug exposures in pSS adults increased the risk for shingles. The risk of shingles varied in pSS adults when exposed to different drugs.

Shingles is known to be more prevalent in patients with autoimmune diseases such as rheumatoid arthritis and SLE [10,11]. In this population-based study, the overall adjusted HR for shingles in pSS patients was 1.69 supporting the hypothesis that pSS is associated with the increased risk of shingles. Nonetheless, the adjusted HR for shingles in pSS is lower than those in patients with either rheumatoid arthritis (2.4) [12] or SLE (2.45) [10] from nationwide population-based data. The universal varicella vaccine was introduced in Taiwan for children born in mid-2002 [10]. All of the subjects in the present study were born before 1990. Furthermore, none of the subjects received zoster vaccines (zostavax) because zostavax was not available prior to 2013 in Taiwan. Thus, the risk of shingles in adult patients with pSS and the control cohorts were not influenced by the varicella vaccine policy in Taiwan and/or zostavax. However, conflicting results with the present study are reported in a retrospective hospital-based study [17]. In the conclusion of that study, the authors mentioned that the risk of shingles in the 17 selected diseases was not compared to that in healthy individuals. It was the major flaw in the design of that study. For example, in assessing the risk of shingles in pSS, the controls were defined as patients with the other 16 diseases. The estimation of risk would be underestimated because most of the other 16 diseases were associated with higher risks of developing shingles [17].

Consistent with data from previous studies [7,28,29], age-specific incidence of shingles in the control cohorts increased with age. Similarly, age-specific incidence of shingles in pSS increased with age and peaked in pSS patients aged 60 and older, indicating that the trend of age-specific incidence in pSS is similar to that of the general population. In age-subgroup analyses of this study, among the adjusted HRs for shingles, the greatest (4.22) was in pSS patients aged 20–29 and the lowest (2.05) was in pSS patients aged 50–59, respectively. Interestingly, similar findings regarding that the adjusted HRs for shingles in pSS tended to decrease with age are also found in SLE patients compared with non-SLE cohorts [10]. Explanations for such findings are made by classifying the risk in one of two categories: the absolute risk and the relative risk. The absolute risk for shingles in pSS patients is represented by age-specific incidence of shingles which increased with age. The findings indicate that old age is a predictor of shingles [7,8]. As for the relative risk, the adjusted HRs for shingles in age-subgroup analyses represent the relative risk for shingles. The risk of shingles is a comprehensive result of immunity linked to the aging process [8,30], comorbidities [18,31], drug exposures [11,18], disease severity [26,32] and nutritional conditions [6,33–35]. The higher relative risk in young adults with pSS could not be attributed to comorbidities and CCI scores [1,18,36] in this study because we used propensity-score matching [31]. In the general population, young adults commonly possess intact immunity and are in good health; whereas, the elderly may have inadequate immune responses due to immunosenescence [6,8,30,37]. The elderly often suffer from comorbidities and are prescribed medications which may impair immunity. In contrast, all pSS patients, including young and elderly, have dysfunctional immunity and diminished health [1,4,36]. Thus, it is reasonable for young pSS patients to have a greater relative risk for shingles. Alternatively, unmeasured residual confounding factors such as disease severity [32], and nutritional deficiencies [6,33–35] are likely to be the causes.

Compared to the control cohorts without steroids/immunosuppressants, pSS adults without drugs had adjusted HRs for shingles 2.06 (95% CI 1.76–2.41), indicating pSS alone is an independent predictor of shingles. The findings support the aspect that pSS is not as benign as traditionally considered [16,38–41]. In general, 40–60% of pSS patients will develop extraglandular symptoms http://www.physio-pedia.com/Sjogren's_Syndrome-cite_note-Monika-3#cite_note-Monika-3 and receive steroids and/or immunosuppressants as the treatment [3]. In this study, we discovered that approximately 49% of pSS patients were prescribed steroids and/or immunosuppressants—consistent with epidemiological findings. The adjusted HRs for shingles in pSS patients receiving steroids alone was 1.27 (95% CI 1.03–1.56), which was close to that in those receiving immunosuppressants alone 1.53 (95% CI 1.08–2.15). Combined therapies of steroids and immunosuppressants resulted in the greatest risk of shingles in pSS patients with the adjusted HR 2.06 (95% CI 1.52–2.79). These results were similar to previous findings that usage of steroids alone, immunosuppressants alone or combined therapy is significantly associated with increased incidence of shingles in patients with rheumatoid arthritis [11]. The overall adjusted HRs for shingles in pSS adults receiving drugs was 1.40 (95% CI 1.16–1.69) compared to pSS adults without drugs. However, compared to pSS adults without drugs, a significant greater risk of shingles in pSS adults receiving drugs was noted in pSS adults aged ≧50 but not in pSS adults aged 20~49. Thus, pSS patients, particularly those receiving medications, should be informed of the increased risk of shingles in order to recognize shingles early and prevent shingles-related complications [32]. In pSS adults aged ≧50, zoster vaccines prior to beginning immunosuppressive drugs might be considered [8].

Similar to adults without pSS, postherpetic neuralgia is the most common complication of shingles in adults with pSS [7]. Shingles-related hospitalization and postherpetic neuralgia in adults with pSS were significantly greater than those in the control cohorts. The findings regarding the higher risk of shingles and postherpetic neuralgia in adults with pSS raises a question—should zostavax be used on pSS patients? Active immunization with zostavax, a live attenuated vaccine, has been shown to be effective in preventing shingles and postherpetic neuralgia in the immunocompetent elderly [8]. But, vaccination of immunocompromised subjects with zostavax is not recommended at present. Recently, two retrospective cohort studies and a small prospective study have demonstrated the safety of Zostavax in selected autoimmune diseases compared with the control cohorts. However, the results are preliminary [42–44]. Further large-scale studies are needed to investigate the safety and efficacy of the zoster vaccination in immunocompromised patients such as pSS and to help refine guidelines for the use of zoster vaccines.

### Strengths and limitations

The present study has four advantages. First, nationwide population-based data including outpatient and inpatient service claims were used to decrease selection bias arising from including only severely affected patients [23]. Second, using propensity-score matching reduced selection bias and confounded associations [24]. Third, the relative homogenous population of Taiwanese residents reduces potential confounding by race because racial differences may be a risk factor for shingles [45]. Fourth, pSS patients were identified by their catastrophic illness certificate, approved by the review committee according to the specific criteria of each diagnosis [1]. Thus, the accuracy of the pSS diagnosis was not a concern.

There are several limitations of this study. First, shingles cases in this retrospective study were defined by diagnostic codes without medical record review [46]. Nonetheless, excellent correlates for the diagnosis of shingles between ICD-9 codes and chart review have been demonstrated [13]. Second, an information bias may have occurred if shingles in pSS patients was preferentially diagnosed because pSS patients had more visits to physicians for their pSS and greater comorbidities. Another information bias may have arisen if pSS patients were more likely to be diagnosed with shingles than patients in the matched control cohorts because physicians were more alert to the diagnosis of shingles in patients with autoimmune diseases [32]. Third, this study did not investigate the risk of shingle in the subgroup of biologic agents alone due to a small number of pSS patients receiving biologic agents. Fourth, this study cannot differentiate the severe effects of the underlying disease from the confounding effects of pharmacological treatments. The complex effects of disease severity and medications affect all study subgroups [26]. However, it is not possible for one study to assess all risk factors and to avoid all confounders. In this study, propensity-score matching was used. All adjusted HRs for shingles from all subgroups of pSS patients demonstrated increased risks of shingles, which validate the findings.

In conclusion, this population-based study showed that pSS adults were at greater risk for shingles and postherpetic neuralgia compared to the control cohorts. Drug exposures increased the risk of shingles significantly. Physicians should be aware of the greater incidence of shingles among adults with pSS and the use of zoster vaccines prior to beginning immunosuppressive drugs in pSS patients might be considered. They should also be familiar with proper antiviral therapy and pain management to reduce the morbidity that ensues from the outbreaks of shingles. Future studies are needed to elucidate the nature of the associations between pSS and shingles and to investigate the safety and efficacy of zoster vaccine in patients with pSS.

## Supporting Information

S1 Appendix A list of ICD-9-CM codes for nonpain complications of shingles. (DOC)

S1 Fig Flowchart showing the numbers of patients who met inclusion and exclusion criteria as well as exposure to different pharmacological treatments. (DOC)
