# Supplementary material for: Risk of Shingles in Adults with Primary Sjogren’s Syndrome and Treatments: A Nationwide Population-Based Cohort Study
Source: PLoS One. 2015 Aug 25;10(8):e0134930. doi: 10.1371/journal.pone.0134930 (PMC4549303; doi:10.1371/journal.pone.0134930)
Supplement: S1 Appendix — (DOC) [file pone.0134930.s001.doc]

**Appendix**

ICD-9-CM codes for nonpain neurological complications of shingles included meningitis (320), cerebellitis (322), encephalitis (323), encephalopathy (348), muscle weakness (728.87), stroke syndrome (v17.1, 438.2, 342) and facial palsy (351). For dermatological complications, cellulites, abscess (680–686), erysipelas (035), pyomyositis, necrotizing fasciitis (728), blepharitis (373, 376.01), scarlet fever and Streptococcal or Staphylococcal infection (034, 041) were included. Complications of others consisted of respiratory and ear complications. Respiratory complications included pneumonia (480–487), pneumonitis (510–519), and bronchitis (466 and 490). Ear complications included otitis externa (053.71) and Ramsay Hunt syndrome (053.1).
