# Supplementary material for: Risk of Shingles in Adults with Primary Sjogren’s Syndrome and Treatments: A Nationwide Population-Based Cohort Study
Source: PLoS One. 2015 Aug 25;10(8):e0134930. doi: 10.1371/journal.pone.0134930 (PMC4549303; doi:10.1371/journal.pone.0134930)
Supplement: S1 Fig — (DOC) [file pone.0134930.s002.doc]

Adults (≧ 20 years old) meeting inclusion criteria (ICD-9-CM 710.2 and approval of the catastrophic illness certificate for Sjogren’s syndrome between 2001 and 2008) (n=8,423)

Study cohort (n=4,287)

No drugs (n=2,177)

Steroids alone (n=1,471)

Immunosuppressants alone (n=335)

Combined therapies (n=304)

Previous shingles (n=444)

Secondary Sjogren’s syndrome (with other autoimmune and/or other connective tissue diseases) (n= 3,474)

Patients with radiotherapy to the head and/or neck, lymphoma, sarcoidosis, hepatitis C viral infection, organ transplants, human immunodeficiency virus infection and chronic obstructive pulmonary disease (n=37)

Unmatchable (n=181)
